# Awareness of age-related change is associated with attitudes toward technology and technology skills among older adults

**DOI:** 10.3389/fpsyg.2022.905043

**Published:** 2022-09-09

**Authors:** Anna Schlomann, Nicole Memmer, Hans-Werner Wahl

**Affiliations:** ^1^Network Aging Research, Heidelberg University, Heidelberg, Germany; ^2^Institute for Educational Sciences, Heidelberg University of Education, Heidelberg, Germany; ^3^Institute of Psychology, Heidelberg University, Heidelberg, Germany

**Keywords:** views on aging, subjective age, internet, ICT, digital, old age, survey study

## Abstract

Despite much research in the context of aging and technology, the role of Views on aging (VoA) for differences in technology use and attitudes among older adults has rarely been studied so far. This study focuses on the associations between a multidimensional measure of VoA and technology use, technology skills, and attitudes toward technology in a sample of older adults (*n* = 369, age range: 65–93 years, 48.2% male). We apply the concept of Awareness of age-related change (AARC) to examine the role of positive (AARC-Gains) and negative (AARC-Losses) self-perceptions of aging. Bivariate and multivariate regression analyses were conducted. The results showed positive associations of AARC-Gains with technology skills and technology attitudes. For AARC-Losses, we identified negative associations with technology skills, technology attitudes as well as general technology use. In contrast, associations between subjective age (SA) and all technology-related measures were non-significant. The results stress the importance to consider multidimensional measures of VoA to gain a better understanding of the associations between an individuals’ experiencing of own aging processes and technology adoption. More research is needed to determine the stability of these findings in other samples and for other kinds of technology use and attitudes.

## Introduction

Given the megatrends echoed in the “gray revolution” as well as the digital transition and its obvious societal transformations, the better understanding of old age and technology use in all its complexity is essential (Huxhold et al., 2020; Pew Research Center, 2021). Also of importance, studies have shown that the “digital divide” between the old and the young, reflecting the impact of both age and cohort, is diminishing more slowly than expected, hence older adults continue to be reluctant to adopt new digital technologies (Friemel, 2016; Hauk et al., 2018; König et al., 2018). Furthermore, older adults are often stereotyped as having less technology skills than younger age groups. This may lead to the incorporation of negative stereotypes about technology relevant abilities into an older person’s self-concept and may generalize to negative views on aging (VoA) at large. As a consequence, older individuals may tend to avoid using digital technology due to stereotype threat, hence the fear of confirming negative stereotypes about their social group (Levy, 2009). However, despite much research in the area of aging and technology, how VoA are associated with technology-related attitudes, skills, and the general use of digital technology has received little empirical consideration in the previous literature.

### Views on aging

The nature of VoA seems multifold and includes perceptions, experiences, and interpretations related to the individual process of growing older. In the past, adults’ awareness about their own aging has been mostly investigated based on “subjective age,” hence asking whether an older person feels younger, older, or the same age than her/his chronological age (Diehl et al., 2021).

However, narrowing down VoA to how old a person feels is limited due to at least three reasons. First, subjective age (SA) addresses only a subset of the phenomena related to VoA and quite a specific one, because it implies that distancing from one’s age is critical. Second, SA comes with uni-dimensionality and recent research has shown that multidimensionality in measuring VoA is important to predict outcomes such as health and well-being (Diehl et al., 2021). Third, multidimensionality may become particular important for predicting outcomes, when positive (gain oriented) as well as negative (loss oriented) sub-dimensions are considered in a balanced way.

We rely in this work on one more recently proposed way to consider the multidimensionality of VoA, i.e., the concept of awareness of age-related change (AARC) introduced by Diehl and Wahl (2010). AARC offers a multidimensional way to measure personal experiences of aging and refers to “a person’s state of awareness that his or her behavior, level of performance, or way of experiencing life has changed as a consequence of having grown older” (Diehl and Wahl, 2010, p. 342). Anchored in the life-span developmental psychology meta-principle that development contains gains and losses across the full life-span (Baltes et al., 2006), its focus lies on whether the experienced changes in various life domains that occur with advancing age are perceived as positive (AARC-Gains) or negative (AARC-Losses). As a new element adding to the previous research on VoA in general and AARC in particular, we examine how AARC-Gains and AARC-Looses are associated with technology-related attitudes, skills, and the general use of digital technology.

### Previous work on VoA and technology

Existing research on the association between VoA and digital technology in late life is remarkably rare. Seifert and Wahl (2018) found that SA, measured by the question “How old do you feel?,” kept a significant predictive role for internet use among older adults even after controlling for a set of confounders. Older adults who felt younger than their chronological age were more often “onliners” than older adults who felt the same age or older than their chronological age. Caspi et al. (2019) demonstrated in an experimental study that older people felt older shortly after using an application on a touchscreen tablet, especially when using an unfamiliar application, than they did before using the technology. The authors explain their finding as a technology-primed evocation of a stereotype threat that resulted *via* heightened SA in feelings of increased identification with the group of “older” individuals and thus the intuitive belief that technology is predominantly made for the young generation.

Another recent study (Köttl et al., 2021) examined reciprocal associations of self-perceptions of aging (SPA), understood as individuals’ subjective views on age-related losses and gains in regard to social, physical, and personal competence and everyday information and communication technologies (EICT). Results showed that low EICT engagement led to more negative SPA related to personal competence 3 years later. In contrast, the direct association between SPA and EICT engagement was not significant. However, the authors did not find evidence suggesting that enhancing positive SPA leads to greater engagement in EICT over time.

These examples provide initial support that more positive VoA may be important for technology use, skills, and attitudes both as a predictor as well as a consequence. Inversely, more negative VoA in general may hinder personal and environmental resources including technology (Diehl and Wahl, 2010).

However, to the best of our knowledge, no study so far considered multidimensionality of VoA in the context of technology. Therefore, we use the multidimensional AARC construct to test in which way experienced gain and loss attributed to aging is associated with technology use, skills, and attitudes. Given the arguments noted above, we hypothesize that AARC gains are positively related to more frequent technology use, more positive attitudes toward digital technology, and better subjective technology skills, while AARC losses are associated with less frequent technology use, more negative attitudes toward digital technology, and poorer subjective technology skills.

## Materials and methods

### Data and study sample

Our analyses are based on data from a multi-topic questionnaire study (online and postal survey) conducted from June to September 2020 with a sample of *n* = 557 individuals living in private households in Germany. The age range among all participants was from 42 to 94 years. Since our focus is on older adults, we restricted our analytical sample on participants 65 years and older (*n* = 402). After excluding cases with missing values variables of analytic relevance the remaining sample size for regression analyses amounted to *n* = 369 participants. Participants were recruited *via* convenience sampling from an existing participant panel (see Diehl et al., 2013) and additional resources (e.g., mailing lists, Senior Centers in the city of Heidelberg). The questionnaire was administered *via* online and post you can find descriptive statistics of the two groups in Supplementary Table S1 of the supplement. Due to the small number of those going the postal way (*n* = 26), we refrained from statistical difference testing. Most obviously, those responding online consistently showed better technology skills and higher use of technology, as could be expected.

Approval for the study was obtained from the Institutional Ethics Review Board of Heidelberg University’s Faculty of Behavioral and Cultural Studies (AZ Wahl 2020 1/1). All participants provided written informed consent. Although data collection took place after the first major wave of the COVID-19 pandemic in Germany (starting in March 2020) in the summer of 2020 with declining COVID-19 rates, we solely rely on testing covariation patterns between VoA and digital technology use as well as attitudes toward technology should not be much affected by the pandemic. Such covariation should not be much affected by the pandemic.

### Measures

#### Views on aging

We used the AARC-Gain and AARC-Loss dimensions measured with the 10-item short-form questionnaire (AARC-10 SF; Kaspar et al., 2019), with higher scores indicating more AARC-Gains/Losses. The AARC-10SF is multidimensional in capturing change across five behavioral domains: Health and physical functioning, cognitive functioning, interpersonal relations, social-cognitive and social–emotional functioning, and lifestyle and engagement. Half of the 10 items assess positive (gains) and half assess negative (losses) perceptions of age-related changes. The item stem is, “With my increasing age, I realize that …” and the response format ranges from 1 (*not at all*) to 5 (*very much*). A sample gain item (INT + domain) is, “… I appreciate relationships and people much more.” A sample loss item (LIFE-domain) is, “… I have to limit my activities.” The conceptually derived two-factor structure building on the gain-loss developmental concept of life-span psychology (Baltes et al., 2006) was confirmed using confirmatory factor analysis and independent samples (Brothers et al., 2019; Kaspar et al., 2019; Sabatini et al., 2021). Still, and somewhat different from previous studies, reliability was rather low particularly in case of AARC-Gains (Cronbach’s *α*: 0.54/0.78; McDonald’s *ω*: =0.55/0.79; see Revelle and Zinbarg, 2009).

In addition, we considered, as previous VoA research did, SA. Felt age was measured in accordance with the bulk of previous research (Pinquart and Wahl, 2021) with a single-item question (“How old do you feel most of the time?”). A proportional discrepancy score between felt age and chronological age was computed to measure SA (subjective age = [felt age − chronological age]/chronological age; Rubin and Berntsen, 2006) in order to “age standardize” SA scores and facilitate their interpretation. A negative proportional discrepancy score indicates a SA that is younger than one’s chronological age, whereas a positive score indicates a SA that is older than one’s chronological age. We replaced outliers, i.e., scores more than three standard deviations below the mean (which is a common cutoff criterion in subjective age research; see Stephan et al., 2020), by a missing value to avoid biased estimates. As a result, three cases were excluded.

#### Technology-related measures

We analyzed the skills in using modern technology devices (i.e., laptop, smartphone, tablet, and the internet; scale: 0 = very bad to 6 = very good) as well as the frequency of use of these devices and the internet (scale: 0 = never to 5 = daily). To obtain a more general analytical perspective, we calculated a mean index for technology skills (Cronbach’s *α*: 0.82, range 0–6) and a sum score for technology use (range 0–20). Cases in which at least 3 out of 4 items were answered were included in the index/score calculation. Regarding attitudes toward technology, we included the12-item Subjective Technology Adaptivity Inventory (STAI; Kamin and Lang, 2013). The STAI assesses the perceived personal adaptivity of technological environments and ranges from 0 to 4 with higher values indicating higher levels of subjective technology adaptivity (Cronbach’s α: 0.92). We only use the overall STAI and not its subscales in the analyses.

#### Control variables

As potential confounders, we included chronological age (in years), gender (1 = male/0 = female), education level (0 = low: no University entrance qualification/1 = high: University entrance qualification) and subjective health status (very good/good/less good [ref.]) in the multivariate analyses.

### Statistical analyses

SPSS version 26 (IBM Statistics, Amos, NY) was used for the analyses. Bivariate and multivariate analyses were conducted to examine the relationships between the subjective experience of aging and technology skills, use, and attitudes. We conducted bivariate regression analyses and multiple linear regression analyses to explore the associations between VoA measures and the technology-related variables. Our hierarchical regression analyses follow three steps: In the first step, only the control variables (i.e., chronological age, gender, education level, health status) were included as predictors (M1). Second, we added the unidimensional VoA indicator of SA to the model (M2), and in the third step, AARC-Gains and AARC-Losses were added to the model (M3). This procedure thus allowed us to test, whether a multidimensional measure such as the AARC-Gains and AAR-Losses has incremental value after controlling for SA and thus improve the prediction model.

## Results

### Descriptive statistics

In total, we analyzed *n* = 369 interviews with participants aged 65 years and older (age range: 65–93 years). The mean age was 72.3 years (SD = 5.68) and 48.2% of participants were male. The education level was rather high (79.9% of participants had higher education) and the majority of participants indicated a “good” (49.3%) health status (38.5% very good, 12.2% less good). The descriptive statistics for the VoA and the technology-related measures are presented in Supplementary Table S1. Participants felt on average 8.79 (SD = 7.46) years younger than their chronological age [proportional discrepancy score (SA) = −0.12 SD = 0.10]; they scored higher on the AARC-Gain dimension than on the AARC-Loss dimension. The mean index for technology skills was 3.37 (SD = 1.12), the sum score for technology use was 14.53 (SD = 4.64) and the mean of STAI was 2.32 (SD = 0.79; see Supplementary Table S1).

### Bivariate results

As shown in Table 1, we rather consistently identified significant correlations regarding the relation between AARC-Gains and AARC-Losses and the technology-related measures in the expected direction. AARC-Gains were positively related to skills, and STAI (but non-significant for the frequency of use measures), while AARC-Losses were negatively associated with these variables (only non-significant for laptop use). A higher proportional difference score in SA was negatively linked with some technology skills (i.e., skills in smartphone and internet use and the index variable) indicating that those feeling younger revealed higher affinity to technology. However, the correlations were non-significant for skills in laptop and tablet use, all general use variables and the STAI. Note that all correlations were in the low to medium range. Still, correlations among AARC-Losses and technology related variables were consistently higher as compared to AARC-Gains and SA. Furthermore, AARC-Gains and AARC-Losses were correlated with chronological age, whereas no significant correlation with gender was observed (see also full correlation matrix of study variables, Supplementary Table S2).

**Table 1:** Correlations between AARC (Gains and Losses), SA and technology-related variables.

Technology-related and demographic variables	AARC-Gains	AARC-Losses	Subjective age
**Technology skills**			
Laptop	0.182^***^	−0.191^***^	−0.077
Smartphone	0.173^**^	−0.262^***^	−0.138^**^
Tablet	0.144^**^	−0.248^***^	−0.050
Internet	0.108^*^	−0.234^***^	−0.111^*^
Skill index	0.189^***^	−0.286^***^	−0.109^*^
**Technology use**			
Laptop	0.077	−0.016	−0.030
Smartphone	0.068	−0.165^**^	−0.063
Tablet	0.024	−0.142^**^	0.041
Internet	0.087	−0.136^**^	−0.006
Sum score	0.089	−0.180^***^	−0.022
STAI	0.196^***^	−0.125^*^	−0.095
Chronological age	−0.112^*^	0.235^***^	0.066
Gender	0.014	0.063	0.034

For the correlation with gender, we calculated the Eta-coefficient.

* *p* < 0.05,

** *p* < 0.01,

*** *p* < 0.001.

### Multivariate results

See Table 2 for the results of the hierarchical regression analyses for technology skills. Together with the control variables, the VoA measures explained a significant amount of variance of technology skills [*F* (8,360) = 15.25, *p* < 0.001; adjusted *R*^2^ = 0.246]. While SA did not have a significant association with technology skills (*p* = 0.642), we identified a positive association for AARC-Gains (*b* = 0.079, *p* < 0.001) and a negative association for AARC-Losses (*b* = −0.076, *p* < 0.001) as expected. All control variables (i.e., chronological age, gender, education, health) also showed a significant association with the skill level (see Table 2).

**Table 2:** Stepwise linear regression analyses to predict technology skills.

	M1		M2		M3	
Predictors	*b* (SE)	Value of *p*	*b* (SE)	Value of *p*	*b* (SE)	Value of *p*
Chronological age	−0.040 (0.009)	0.000	−0.039 (0.009)	0.000	−0.027 (0.009)	0.004
Male (ref. female)	0.507 (0.106)	0.000	0.509 (0.106)	0.000	0.543 (0.102)	0.000
Education level: high (ref. low)	0.483 (0.134)	0.000	0.505 (0.135)	0.000	0.464 (0.131)	0.000
Health status: very good (ref. less good)	0.823 (0.175)	0.000	0.759 (0.182)	0.000	0.480 (0.189)	0.011
Health status: good (ref. less good)	0.566 (0.170)	0.001	0.534 (0.171)	0.002	0.377 (0.170)	0.028
SA			−0.691 (0.541)	0.203	−0.261 (0.532)	0.624
AARC-Gains					0.079 (0.019)	0.000
AARC-Losses					−0.076 (0.018)	0.000
Model fit	*F* (5,363) = 17.73, *p* < 0.001		*F* (6,362) = 15.07, *p* < 0.001		*F* (8,360) = 15.25, *p* < 0.001	
Adjusted *R*^2^	0.185		0.187		0.246	

The variable technology skills is calculated as a mean index of different specific technology skills (i.e., skills in using a laptop, smartphone, tablet, and the internet); SA is considered as proportional discrepancy score between felt age and chronological age: subjective age = [felt age − chronological age]/chronological age.

Table 3 shows the results of the same regression analyses for the STAI. Again, the VoA measures and the control variables explained a significant amount of variance in the full model [*F* (8,360) = 6.73, *p* < 0.001; adjusted *R*^2^ = 0.111]. SA did not have a significant association with STAI (*p* = 0.321) but again, AARC-Gains (*b* = 0.062, *p* < 0.001) and AARC-Losses (*b* = −0.032, *p* = 0.024) showed a significant association in the expected direction. Gender also showed a significant association with STAI, but none of the other control variables remains significant in the full model (see Table 3).

**Table 3:** Stepwise linear regression analyses to predict STAI.

	M1		M2		M3	
Predictors	*b* (SE)	Value of *p*	*b* (SE)	Value of *p*	*b* (SE)	Value of *p*
Chronological age	−0.001 (0.007)	0.935	0.000 (0.007)	0.982	0.006 (0.007)	0.378
Male (ref. female)	0.373 (0.080)	0.000	0.374 (0.080)	0.000	0.392 (0.078)	0.000
Education level: high (ref. low)	0.049 (0.101)	0.632	0.066 (0.102)	0.515	0.042 (0.100)	0.673
Health status: very good (ref. less good)	0.308 (0.132)	0.020	0.254 (0.137)	0.064	0.146 (0.144)	0.314
Health status: good (ref. less good)	0.162 (0.128)	0.207	0.135 (0.129)	0.296	0.078 (0.130)	0.550
SA			−0.576 (0.408)	0.159	−0.404 (0.406)	0.321
AARC-Gains					0.062 (0.014)	0.000
AARC-Losses					−0.032 (0.014)	0.024
Model fit	*F* (5,363) = 5.66, *p* < 0.001		*F* (6,362) = 5.06, *p* < 0.001		*F* (8,360) = 6.73, *p* < 0.001	
Adjusted *R*^2^	0.060		0.062		0.111	

SA is considered as proportional discrepancy score between felt age and chronological age: subjective age = [felt age − chronological age]/chronological age.

In Table 4, the results of the regression analyses for the sum score of technology use are presented. In the full model, a significant amount of variance is explained by the VoA measures and the control variables [*F* (8,360) = 8.19, *p* < 0.001; adjusted *R*^2^ = 0.135]. AARC-Losses showed a significant association with the frequency of use in the expected direction (*b* = −0.161, *p =* 0.037) but neither did AARC-Gains (*p* = 0.140) nor SA (*p* = 0.697). Among the control variables, we identified significant associations for chronological age, gender, and education but not for subjective health status (see Table 4).

**Table 4:** Stepwise linear regression analyses to predict technology use.

	M1		M2		M3	
Predictors	*b* (SE)	Value of *p*	*b* (SE)	Value of *p*	*b* (SE)	Value of *p*
Chronological age	−0.179 (0.039)	0.000	−0.179 (0.039)	0.000	−0.157 (0.040)	0.000
Male (ref. female)	0.988 (0.437)	0.024	0.988 (0.437)	0.024	1.054 (0.436)	0.016
Education level: high (ref. low)	2.289 (0.552)	0.000	2.290 (0.557)	0.000	2.216 (0.556)	0.000
Health status: very good (ref. less good)	1.614 (0.717)	0.025	1.610 (0.747)	0.032	1.007 (0.805)	0.212
Health status: good (ref. less good)	1.103 (0.697)	0.114	1.101 (0.705)	0.119	0.754 (0.726)	0.299
SA			−0.041 (2.228)	0.985	0.883 (2.27)	0.697
AARC-Gains					0.117 (0.079)	0.140
AARC-Losses					−0.161 (0.079)	0.037
Model fit	*F* (5,363) = 11.87, *p* < 0.001		*F* (6,362) = 9.86, *p* < 0.001		*F* (8,360) = 8.19, *p* < 0.001	
Adjusted *R*^2^	0.129		0.126		0.135	

SA is considered as proportional discrepancy score between felt age and chronological age: subjective age = [felt age − chronological age]/chronological age.

## Discussion

Considering the multidimensionality of VoA might be important for the better understanding of the relationship between technology related variables and VoA in later life. We applied the concept of AARC-Gains and AARC-Losses to test this assumption. Our results suggest that older adults’ technology skills and attitudes toward technology are not only related to chronological age, as has been shown in previous research (Huxhold et al., 2020; Pew Research Center, 2021), but also to their views of their own aging. In particular, our results based on multivariate analysis show that AARC-Gains are associated with more positive attitudes toward technology and better subjective technology skills, whereas AARC-Losses were related to more negative attitudes and worse subjective technology skills. Beyond that, AARC-Losses were also related to less frequent technology use. In contrast, associations between the technology-related variables and SA were smaller (and non-significant for some variables) in the bivariate analyses and consistently non-significant in hierarchical regressions.

In addition, comparing the results for technology use, skills, and attitudes (measured *via* the STAI), our results suggest that technology skills are more strongly related to VoA than technology attitudes or the frequency of use, because the explained amount of variance (interpretable as effect size) was clearly higher for technology skills. However, more research is needed that will focus on other aspects of technology attitudes. One important extension might be to focus on technology acceptance, understood as the intention to use technologies (Venkatesh and Davis, 2000; Venkatesh and Bala, 2008). Furthermore, other aspects of technology use might be considered, e.g., the variability of internet use (Seifert et al., 2020), to gain a better understanding of the associations between VoA and technology adoption in old age. These further analyses may help to determine whether our findings also hold true for other dimensions of technology attitudes and for a more refined analysis of technology use.

Of note, better educated older adults, although they used technology more often and were also more confident in their technology skills, did not show a more positive attitude toward technology. Previous work by Wasserman and Richmond-Abbott (2005) suggests that more frequent use of technology and better perceived skill level does not necessarily generalize to technology related attitudes.

We conclude that a multidimensional approach of VoA is indeed more informative than going solely uni-dimensionally. Our results are consistent with other research demonstrating that negative self-perceptions of aging are associated with lower engagement in everyday information and communication technologies (Köttl et al., 2021). In contrast, our results differ from those reported by Seifert and Wahl (2018) who found significant associations between SA and technology-related measures. This stresses the importance of further research in the context of VoA and technology and the need for multidimensional assessments to gain more insights into their associations.

Furthermore, our findings add to the discussion of stereotype threat in the context of technology use. When more negative VoA go hand in hand with worse technology skills and less positive attitudes toward technology, this may be an indication for the already existing incorporation of negative stereotypes about technology use among older adults and may negatively affect their intentions to use new technological devices. As a consequence, this would manifest and maybe even enlarge the “gray divide” (Quan-Haase et al., 2018).

### Limitations

Our sample was not representative and participants with high education, good (subjective) health status, better technology skills, and higher technology use were overrepresented in our sample. Replication studies in representative samples are therefore necessary. In particular, it needs future research to determine whether the observed associations among indicators of VoA and technology parameters also hold in less advantaged samples. Further, the rather low item correlations for the AARC-Gains scale may reflect the purposeful and conceptually driven use of heterogeneous items particularly in the Gains subscale, hence including quite specific gains in domains mostly seen as indicating loss such as physical and cognitive functioning. We were only able to analyze associations among study variables based on cross-sectional data. Longitudinal data would allow to analyze in more detail whether VoA are a predictor or a consequence of technology use, skills and attitudes and further research is needed to gain more insights into these associations. Finally, although inter-relations were rather consistent, they were limited in magnitude both the bivariate level as well as in terms of explained variance in regression analyses.

### Conclusion

This research reports new insights into the associations between technology-related variables and different measures of VoA with a focus on the two dimensions of AARC-Gains and AARC-Losses. Findings stress the importance to consider multidimensional measures of VoA. We also interpret our findings as initial evidence supporting that technology-enhancing education and training may integrate the role of VoA as an element of their curriculum.

## Data availability statement

The raw data supporting the conclusions of this article will be made available by the authors, without undue reservation.

## Ethics statement

The studies involving human participants were reviewed and approved by Institutional Ethics Review Board of Heidelberg University’s Faculty of Behavioral and Cultural Studies. The patients/participants provided their written informed consent to participate in this study.

## Author contributions

All authors listed have made a substantial, direct, and intellectual contribution to the work and approved it for publication.

## Funding

The article processing charge was funded by the Baden-Württemberg Ministry of Science, Research, and Culture and the Heidelberg University of Education in the funding programme Open Access Publishing.

## Conflict of interest

The authors declare that the research was conducted in the absence of any commercial or financial relationships that could be construed as a potential conflict of interest.

## Publisher’s note

All claims expressed in this article are solely those of the authors and do not necessarily represent those of their affiliated organizations, or those of the publisher, the editors and the reviewers. Any product that may be evaluated in this article, or claim that may be made by its manufacturer, is not guaranteed or endorsed by the publisher.
