# Supplementary material for: Awareness of age-related change is associated with attitudes toward technology and technology skills among older adults
Source: Front Psychol. 2022 Sep 9;13:905043. doi: 10.3389/fpsyg.2022.905043 (PMC9505520; doi:10.3389/fpsyg.2022.905043)
Supplement: Supplementary file 1 [file Data_Sheet_1.docx]

SUPPLEMENT

Table S1

Descriptive Statistics of AARC (Gains and Losses), subjective age and technology-related variables, depending on assessment procedure (online versus postal)

|  | Total *M (SD)/ %* | *n* | Online *M (SD)/ %* | *n* | Postal *M (SD)/ %* | *n* |
| --- | --- | --- | --- | --- | --- | --- |
| Chronological age  Range (years) | 72.3 (5,68)  65-93 | *369* | 72,01  65-91 | *343* | 76.04 (8.15)  65-93 | *26* |
| Male | 48.2 % | *178* | 50.1 % | *172* | 23.1 % | *6* |
| Female | 51.8 % | *191* | 49.9% | *171* | 76.9 % | *20* |
| Education level: high | 79.9 % | *295* | 82.8 % | *284* | 42.3 | *11* |
| Education level: low | 21.1 % | *74* | 17.2 % | *59* | 57.7 | *15* |
| Health status: very good | 38.5 % | *142* | 38.8 | *133* | 34.6 % | *9* |
| Health status: good | 49.3 % | *182* | 49.3 | 169 | 50 % | *13* |
| Health status: less good | 12.2 % | *45* | 12.0 | *41* | 15.4 % | *4* |
| ***Views on Aging*** |  |  |  |  |  |  |
| Felt Age – chronological age | -8.79 (7.46) | 369 | -8.49 (7.27) | 343 | -12.73 (8.83) | 26 |
| SA^1^ | -0.12 (0.10) | 369 | -0.12 (0.10) | 343 | -0.17 (0.12) | 26 |
| AARC-Gains^2^ | 18.57 (2.80) | 369 | 18.62 (2.79) | 343 | 17.96 (2.88) | 26 |
| AARC-Losses^2^ | 11.14 (2.28) | 369 | 11.11 (3.24) | 343 | 11.46 (3.77) | 26 |
| ***Technology Skills^3^*** |  |  |  |  |  |  |
| Laptop | 3.37 (1.42) | 367 | 3.49 (1.30) | 341 | 1.77 (1.88) | 26 |
| Smartphone | 3.33 (1.41) | 369 | 3.44 (1.32) | 343 | 1.85 (1.74) | 26 |
| Tablet | 2.83 (1.69) | 364 | 2.93 (1.65) | 339 | 1.56 (1.80) | 25 |
| Internet | 3.97 (0.97) | 369 | 4.06 (0.83) | 343 | 2.77 (1.66) | 26 |
| Skill Index^4^ | 3.37 (1.12) | 369 | 3.48 (1.02) | 343 | 2.01 (1.54) | 26 |
| ***Technology Use ^5^*** |  |  |  |  |  |  |
| Laptop | 3.24 (2.17) | 366 | 3.35 (2.12) | 341 | 1.64 (2.27) | 25 |
| Smartphone | 4.20 (1.75) | 368 | 4.32 (1.62) | 342 | 2.62 (2.48) | 26 |
| Tablet | 2.36 (2.29) | 368 | 2.45 (2.29) | 342 | 1.19 (2.00) | 26 |
| Internet | 4.79 (0.80) | 369 | 4.90 (0.45) | 343 | 3.31 (2.04) | 26 |
| Technology Use Score^6^ | 14.53 (4.64) | 369 | 14.98 (3.89) | 343 | 8.69 (6.89) | 26 |
| ***STAI^7^*** | 2.32 (0.79) | 369 | 2.38 (0.75) | 343 | 1.58 (0.91) | 26 |

Note: ^1^SA is considered as proportional discrepancy score between felt age and chronological age: subjective age = [felt age − chronological age] / chronological age. ^2^scale = 5-25; higher scores indicating more AARC-Gains/Losses. ^3^scale: 0 = very bad to 6 = very good. ^4^mean index range 0 = very bad to 6 = very good. ^5^scale: 0 = never to 5 = daily. ^6^ sum score range 0 to 20; with higher scores indicating more frequent use. ^7^ ranges from 0 to 4 with higher values indicating higher levels of subjective technology adaptivity.

Table S2: Correlations of all study variables (Pearson-Correlation)

|  | Lap-  top use | Smart-  phone use | Tablet use | Inter-  net use | Techno-  logy Use Score | Laptop skills | Smart-phone skills | Inter-  net skills | Tablet skills | Skill Index | STAI | SA | AARC-  Gains | AARC-  Losses | Age | Edu-  cation | Health |
| --- | --- | --- | --- | --- | --- | --- | --- | --- | --- | --- | --- | --- | --- | --- | --- | --- | --- |
| Laptop use | 1 | ,146^**^ | ,058 | ,288^***^ | ,628^***^ | ,498^***^ | ,105^*^ | ,157^**^ | ,075 | ,256^***^ | ,108^*^ | -,030 | ,077 | -,016 | -,050 | ,177^**^ | -,042 |
| Smart-  phone use | ,146^**^ | 1 | ,254^***^ | ,269^***^ | ,648^***^ | ,200^***^ | ,738^***^ | ,181^***^ | ,327^***^ | ,460^***^ | ,266^***^ | -,063 | ,068 | -,165^**^ | -,259^***^ | ,167^**^ | -,144** |
| Tablet use | ,058 | ,254^***^ | 1 | ,179^**^ | ,668^***^ | ,206^***^ | ,309^***^ | ,190^***^ | ,614^***^ | ,430^***^ | ,196^***^ | ,041 | ,024 | -,142^**^ | -,182^***^ | ,082 | -,127* |
| Internet use | ,288^***^ | ,269^***^ | ,179^***^ | 1 | ,528^***^ | ,416^***^ | ,277^***^ | ,531^***^ | ,270^***^ | ,431^***^ | ,267^***^ | -,006 | ,087 | -,136^**^ | -,221^***^ | ,265^***^ | -,026 |
| Use Score | ,628^***^ | ,648^***^ | ,668^***^ | ,528^***^ | 1 | ,507^***^ | ,555^***^ | ,353^***^ | ,528^***^ | ,609^***^ | ,314^***^ | -,022 | ,089 | -,180^**^ | -,268^***^ | ,242^***^ | -,146** |
| Laptop skills | ,498^***^ | ,200^***^ | ,206^***^ | ,416^***^ | ,507^***^ | 1 | ,492^***^ | ,641^***^ | ,564^***^ | ,821^***^ | ,456^***^ | -,077 | ,182^***^ | -,191^***^ | -,142^**^ | ,168^**^ | -,127* |
| Smart-  phone skills | ,105^*^ | ,738^***^ | ,309^***^ | ,277^***^ | ,555^**^ | ,492^***^ | 1 | ,517^***^ | ,602^***^ | ,808^***^ | ,490^***^ | -,138^**^ | ,173^**^ | -,264^***^ | -,283^***^ | ,148^**^ | -,253*** |
| Internet skills | ,157^**^ | ,181^***^ | ,190^***^ | ,531^***^ | ,353^***^ | ,641^***^ | ,517^***^ | 1 | ,522^***^ | ,775^***^ | ,476^***^ | -,111^*^ | ,108^*^ | -,234^***^ | -,190^***^ | ,194^***^ | -,169** |
| Tablet skills | ,075 | ,327^***^ | ,614^***^ | ,270^***^ | ,528^***^ | ,564^***^ | ,602^***^ | ,522^***^ | 1 | ,855^***^ | ,473^***^ | -,050 | ,144^**^ | -,248^***^ | -,202^***^ | ,152^**^ | -,221*** |
| Skill Index | ,256^***^ | ,460^***^ | ,430^***^ | ,431^***^ | ,609^***^ | ,821^***^ | ,808^***^ | ,775^***^ | ,855^***^ | 1 | ,575^***^ | -,109^*^ | ,189^***^ | -,286^***^ | -,251^***^ | ,199^***^ | -,239*** |
| STAI | ,108^*^ | ,266^***^ | ,196^***^ | ,267^***^ | ,314^***^ | ,456^***^ | ,490^***^ | ,476^***^ | ,473^***^ | ,575^***^ | 1 | -,095 | ,196^***^ | -,125^*^ | -,027 | ,111^*^ | -,144** |
| SA | -,030 | -,063 | ,041 | -,006 | -,022 | -,077 | -,138^**^ | -,111^*^ | -,050 | -,109^*^ | -,095 | 1 | ,013 | ,309^**^ | ,066 | ,112^*^ | ,293*** |
| AARC-  Gains | ,077 | ,068 | ,024 | ,087 | ,089 | ,182^***^ | ,173^**^ | ,108^*^ | ,144^**^ | ,189*^**^ | ,196*^**^ | ,013 | 1 | ,114^*^ | -,112^*^ | ,067 | ,012 |
| AARC-  Losses | -,016 | -,165^**^ | -,142^**^ | -,136^**^ | -,180^**^ | -,191^***^ | -,264^***^ | -,234^***^ | -,248^***^ | -,286^***^ | -,125^*^ | ,309^***^ | ,114^*^ | 1 | ,235^***^ | -,018 | ,446*** |
| Age | -,050 | -,259^***^ | -,182^***^ | -,221^***^ | -,268^***^ | -,142^**^ | -,283^***^ | -,190^***^ | -,202^***^ | -,251^***^ | -,027 | ,066 | -,112^*^ | ,235^***^ | 1 | -,091 | ,161** |
| Edu-  cation^1^ | ,177^**^ | ,167^**^ | ,082 | ,265^***^ | ,242^***^ | ,168^**^ | ,148^**^ | ,194^***^ | ,152^**^ | ,199^***^ | ,111^*^ | ,112^*^ | ,067 | -,018 | -,091 | 1 | -,027 |
| Health^2^ | -,042 | -,144** | -,127* | -,026 | -,146** | -,127* | -,253*** | -,169** | -,221*** | -,239*** | -,144** | ,293*** | ,012 | ,446*** | ,161** | -,027 | 1 |

Note: *p<.05, **p<.01, ***p<.001; ^1^scale: 0 = Education level low to 4 = Education level high; ^2^scale: 0 = bad to 5 = very good
